# Russian nurses’ readiness for transcultural care of palliative patients

**DOI:** 10.1186/s12904-023-01198-1

**Published:** 2023-07-05

**Authors:** Nataliya Kasimovskaya, Natalia Geraskina, Elena Fomina, Svetlana Ivleva, Maria Krivetskaya, Nina Ulianova, Marina Zhosan

**Affiliations:** Department of Nursing Management and Social Work, Sechenov First Moscow State Medical University (Sechenov University), Moscow, Russian Federation

**Keywords:** Palliative care, Medical staff, Patient care, Transcultural nursing

## Abstract

Palliative care involves an approach aimed at improving the quality of life of patients and their families, who are forced to cope with the problems associated with life-threatening diseases. This definition includes a growing group of patients around the world. It requires an extension of the definition of patients in need of palliative care in countries such as Russia and a significant improvement in the work of nursing personnel with these patients. This study aims to determine the level of preparedness of nursing personnel for specialized care (transcultural care) and the quality of care provided to palliative patients. The presented findings of the study demonstrate the relevance of developing transcultural competence, which enables significant improvement in the quality of life of palliative patients. The analysis of medical workers’ assessment of the level of specific training and their intercultural preparedness was conducted based on hospices (Moscow). A survey was conducted among 113 medical workers of the middle level of education aged between 28 and 56 (average of 44.2 years) and experience in palliative care ranged from 3 to 18 years (average of 9.5 years). The Intercultural Readiness Check (IRC) test, widely used to assess nursing staff worldwide, was used for the survey to determine the level of readiness for transcultural care. A strong correlation was found between a number of the test scales and measures of participants’ age and experience. The presented material demonstrates the realization of an interdisciplinary approach to the issues of specific training of nursing personnel in the field of “transcultural care” in providing palliative care to incurable patients.

## Introduction

The importance of transcultural care in the preparation and nurses’ readiness is determined by the multinational nature that characterizes many countries. The ethnic composition of the Russian population, according to the 2010 census results (following the methodology adopted during the census), has 192 national groups, including a significant number of cultural-historical and ethnic subgroups. Like most countries of the modern world, the Russian Federation is undergoing depopulation. According to census data, the working-age population decreased by 959,000 people (1.1%) from 2002 to 2010. Half of the working-age population is over 35 years old. The median age was 35 for men, 41 for women, and 38 for the population as a whole. There has been a shift in the specific weight of the population toward the age of 40–60 years for both sexes [1]. The diversity of the country’s ethnic composition is also accompanied by considerable linguistic diversity: according to census data, 170 languages are spoken in the Russian Federation. Based on census results and sociological studies, over 60 religions are practiced in the country. Among them, all the main directions of world religions are represented, as well as most of the principal branches of Protestantism, pagan and neopagan beliefs, modern religious movements, and more [1].

The necessity of improving nursing care for aged patients is a response to the challenge of age-related poly morbidity (comorbidity). The problem of rising morbidity rates in the population, particularly the prevalence of poly morbidity in clinical cases and the resulting increase in patients with incurable diseases, is a pressing issue in Russia as well as in many other countries. In modern usage, it refers to the combination of two or more chronic diseases in the same patient. There are a few methods for assessing poly morbidity. The most commonly recognized is the Charlson poly morbidity index [2]. There is a significant problem in that studies are conducted in very different population samples and research numbers regarding the prevalence of poly morbidity differ significantly [3]. Polymorbidity is one of the most urgent, complex, and multifaceted problems, which has clinical, social, and economic importance.

Depopulation is also a factor that significantly increases the demand for palliative care nurses and the requirements for their training. Today, practically all developed countries and the majority of developing countries suffer from depopulation [4]. This phenomenon is leading to a rapidly increasing need for care for the elderly. There is a growing number of people with incurable diseases among them.

Palliative care (from Latin pallium - covering, cloak) is an approach that improves the quality of life of incurable patients. The consensus-based definition of palliative care agreed upon by most researchers is as follows: “Palliative care is the active holistic care of individuals across all ages with serious health-related suffering due to severe illness and especially of those near the end of life. It aims to improve the quality of life of patients, their families, and their caregivers” [5]. Today there is a significant increase in the number of palliative patients all over the world. In its turn, palliative medicine is part of palliative care. The tasks of palliative medicine are to use new methods and combine modern world achievements to correct those or other chronic conditions when opportunities for radical treatment have been exhausted. Legislative frameworks, associations, medical organizations, and volunteer movements to assist palliative patients are being actively developed to address these issues [6–8].

The key actor in palliative care is the nurse. The theory of a nurse’s role in palliative care rightfully belongs to Virginia Henderson [9], whose works reflect the most important stages of knowing a palliative patient and the influence of proper care on the quality of life of an incurable patient. Henderson’s theories have subsequently become axiomatic in the care of palliative patients. These provisions have not lost their relevance until today [10, 11].

The high quality of palliative care can be ensured by a multidisciplinary team, which should be formed of medical specialists of higher and middle level, representatives of the clergy, social services, etc. At the same time, a nurse is the core of this team, and their tasks are coordination of all specialists, integration of all recommendations, and adaptation to a particular patient. These tasks include defining a patient’s problems, principles of general care, psychological support not only for a patient but also for their relatives, and symptom control. Therefore, the quality of this care depends on the ability to properly prioritize, competently develop a care plan, and implement it [12–14].

The system of palliative care varies from country to country. It depends on the financing and social level of each country, the possibility of training nursing staff and providing the exchange of experience with representatives of this service from other countries, and the level of compensation for the personnel working in the structure of palliative care. However, of great importance is what is known as transcultural nursing (a specific nursing specialty that is closely related to the concepts of ethnic culture and comparative cultural care, which emerged around 1955 as a formal field of practice and in 1988 was recommended for use in research or track programs by Transcultural Nursing Society) [11, 15, 16].

### Literature review

Transcultural nursing is a humanitarian discipline whose task is to serve people, organizations, and societies defined within the context of culture as competent nursing is considered to be based on knowledge of cultural values. The Transcultural Nursing Society has established the following goals:

- to promote the cultural competence of the nursing society worldwide;
- to disseminate the substantive knowledge necessary to put cultural competence into practice;
- develop strategies to advocate for social change for effective nursing;
- to be a financially non-profit entity [17].

Cultural competence involves understanding the religious values and traditions of different ethnic, religious, or social groups. Increasingly, cultural humility is being considered alongside cultural competence. It involves the ability of the individual to recognize their limitations to avoid errors in judgment and action concerning other cultures; this also includes the ability to understand the strengths and weaknesses of one’s own culture and work respectfully with people from diverse cultures [18, 19]. Health literacy is an area of knowledge in which people can assimilate medical information to ensure a decent quality of life, including for chronically progressive diseases [20, 21]. It has been statistically proven that knowledge of a patient’s traditions and worldview and the establishment of a trusting relationship at the pre-treatment stage yield higher rates of the treatment process [22–24].

Transcultural nursing care today is an important aspect of health care policy in developed countries. Despite the rapid improvement in the quality of care, the continuing diversity of the multicultural society in the United States poses a major challenge to nurses providing individual or complex care to patients, necessitating a cultural awareness of not only the nation as a whole but also of each individual’s values. The task of a nurse is to make a culturally competent nursing plan, which should be based on the analysis of the learned knowledge in this field and compare it with the aspect of real practice. However, additional nursing research is needed to facilitate the further development of transcultural nursing, which requires additional resources, public, and state support. According to WHO, the UK spends about 2% of the research budget on palliative care, and in the United States, at least 1% of the budget is devoted to palliative care [25, 26].

Special programs targeting specific ethnic groups of patients have been developed in different countries. The introduction of a nursing care model for an education program based on Islamic teachings and practices has subsequently led to significant improvements in the quality of care provided [27–29]. The level of cultural competence had a causal relationship with the development of multiple organ failure and premature death. Understanding of gastronomic culture of this or that community is also an integral part of nursing transcultural care [30, 31].

Campinha-Bacote’s Model for Nurses has been developed to provide culturally competent care to patients of the Hispanic ethnic group. This model, which strategy is based on the ASKED (Awareness, Skills, Knowledge, Encounters, and Desire) principle, incorporates a system for assessing meaningful cultural characteristics of patients and involves simultaneously providing health literacy to people with lower social awareness [15]. Thus, nurses, in parallel to caring for ethnic minorities, compensate for sanitary illiteracy in some communities and increase their cultural competence. Research results revealed a predominant lack of or insufficient cultural competence and the need to improve it by incorporating cultural values into the nursing curriculum [32–34].

To improve the quality of primary health care delivery to immigrants in Canada, a multifactorial study was conducted, which included three groups of factors in a single comprehensive approach:

1. an individual-level approach (improving self-esteem about one’s health, communication skills, and knowledge of cultural differences in health care);
2. community-based approach (health education sessions, support programs in various routine situations);
3. approach to the level of care included taking the necessary steps to ensure an effective doctor-patient relationship (inviting cross-cultural staff to collaborate to gain cultural skills).

This study also highlighted the need to engage policymakers to shape new programs and promote the construction of additional medical facilities and diagnostic centers to ensure high-quality care [35–37]. Questions of measuring transcultural competence and its various components have been studied for decades [17, 38, 39]. In several cases, well-known and frequently used tests of transcultural readiness and the level of transcultural communication have a high level of correlation with each other, which allows them to be used either simultaneously or to clarify certain indicators [18, 40]. A significant number of tests of multicultural competence in health care and nursing are translated into other languages and undergo additional testing [41]. In a number of cases authors of academic questionnaires for the study of transcultural competence point out that the presence of such competence correlates with the personal development of medical workers and that this competence contributes to their formation as a personality [42–44].

### Problem statement

Proceeding from the topical aspects of palliative care, the work aimed to determine the level of preparedness of nurses working in the Russian Federation for transcultural care of palliative patients.

## Methods and materials

### Participants

An analysis of the specific training of 113 nurses was conducted to achieve the research objective. The study participants included mid-level medical (nursing) staff of the following categories: ward (post) nurse, procedure nurse, dressing nurse, massage nurse, senior nurse, and head nurse. Interviewed medical staff represented the following types of organizations and subdivisions of medical institutions: adult palliative care unit, adult hospice, and adult nursing unit. The survey was conducted in 6 different medical institutions in Moscow of the above-mentioned areas for the period 2019–2021. The selection of participants was based on lists of nursing staff of the corresponding profile and random sampling. All survey participants were women, aged between 28 and 56 (average 44.2 years). The selection was not based on gender, but the random sampling included only women, which may indicate a certain gender imbalance in the profession. Study participants’ work experience in palliative care ranged from 3 to 18 years (an average of 9.5 years). The normality of the distribution of age and experience values in this sample about the general sample was checked using the Shapiro-Wilk test. The results are shown in Table 1.

**Table 1:** Results of the Shapiro-Wilk test to test the hypothesis of normal distribution of age and experience in a sample of participan

	*W*	*p*
Age	0.918	0.001
Experience	0.837	0.001

Based on the results presented in Table 1, it is impossible to reject the hypothesis of a normal distribution. Presumably, the distribution of age and palliative care experience in the sample was close to normal. Therefore, the survey results did not require special corrections for the unevenness of the statistical distribution on these criteria.

### Research design

The survey was conducted in the form of a face-to-face survey when participants filled out The Intercultural Readiness Check (IRC) questionnaire on their own [40]. The Intercultural Readiness Check is one of the most widely known and used tests for the study of intercultural competence in organizations and companies in many countries of the world. The test is also widely used in nursing. The validity and reliability of the test were tested by researchers many times [44]. The test used was examined for construct validity against an existing instrument for multicultural effectiveness: The Multicultural Personality Questionnaire (MPQ). Research has shown that the instrument was equally strongly related to international inspiration, open-mindedness (MPQ), and intercultural sensitivity (IRC) as the most important predictors. The scales of the IRC questionnaire had an advantage over other tests since more variance in international experience was explained [40]. Reliability was confirmed across groups of respondents from different countries [44].

The questionnaire was translated into Russian by a professional translator and then translated from Russian into English by another translator. A secondary back-translation was compared with the original. The final Russian version of the questionnaire was determined with the help of both translators. In this case, the main research method is the analysis of descriptive statistics. The study aims to establish the level of Russian nurses’ readiness for transcultural practice in palliative care.

It was also important to check whether there were any correlations between the age and experience of the participants and their scores on each of the IRC scales. This correlation study aims to determine whether there is a relationship between the age and experience of nurses and their readiness for intercultural care. For this purpose, Pearson’s direct correlation method was used. The study results are presented in Table 2.

**Table 2:** The Intercultural Readiness Check (IRC) tests scales correlation with the age and experience of nursing staff (Pearson’s correlation used)

Scale	Age	Experience
Intercultural sensitivity	0.146	0.194
Intercultural communication	0.268	0.284
Intercultural relation building	0.129	0.108
Conflict management	0.386	0.433*
Leadership	0.477*	0.412*
Tolerance of ambiguity	0.816*	0.782*

Significance level: p < .05. * correlation strength can be considered high

The scope of palliative care included: the identification of a patient’s problems and priority symptom complex, control over it, general care, and actions according to the psychological features of a palliative patient and their relatives. Communicating with patients and their relatives is a kind of bonding tool for creating quality palliative care and trusting relationships, which requires several specialized skills.

To obtain a reverse assessment of the need for transcultural care, a survey of patients’ relatives was conducted. The need for this survey is due to the motivation of the study: only if transcultural care is a need of patients articulated by their relatives, it becomes necessary to investigate the level of this skill in palliative care nurses. The sample was made among relatives of the patients who received care from the study nurses-participants during the study. A total of 32 relatives of patients were surveyed, representing six ethnic groups from 4 regions of the Russian Federation. This non-representative group was used for further monitoring of the research results and for selecting comments regarding the work of medical personnel. Participants were asked to rate on a five-point Likert scale (from 1 - “not at all important” to 5 - “very important”) the importance for them of health care staff taking their culture into account when they are in palliative care units. Patients were aware that cultural sensitivity, gastronomic culture, and knowledge of a patient’s language were included in psychosocial support according to their cultural norms.

### Statistical processing

Preparation and processing of statistical data were performed in MS Excel, and subsequent analysis - in the application package IBM SPSS Statistics Version 20.0.

### Research limitations

The study covers only a subset of the diverse ethnic and cultural groups present in the Russian Federation who need palliative care. It should be noted in mind that other large urban centers in Europe, America, and other countries may contain a much larger ethnic representation. The usage of a questionnaire and subjective assessment to evaluate the tool, rather than objective observation or expert evaluation of care, can be considered a limitation. This may be one of the aims of future research.

### Ethics approval

The research was conducted ethically by the World Medical Association Declaration of Helsinki. The research was approved by the local ethics committees of Sechenov First Moscow State Medical University (Sechenov University) (Protocol No. 3929 dated from 02/02/2019). Participants provided informed consent.

## Results

The obtained test results significantly differ on separate scales but are generally close to the mean values, considering that each separate scale is evaluated on a 5-point Likert scale. According to the results presented in Table 3, it is noticeable that the mean values on the scales assessing intercultural sensitivity and communication are below the average (2.5 points) (2.31 and 2.18, respectively). For the other scales, the mean scores are above average. This may indicate that nurses are aware of a number of significant intercultural communication skills as being below a desirable level.

**Table 3:** Results of The Intercultural Readiness Check (IRC) test

Scale	M	SD	Median
Intercultural sensitivity	2.31	0.31	1.89
Intercultural communication	2.18	0.39	1.78
Intercultural relation building	2.59	0.29	2.51
Conflict management	3.22	0.4	2.82
Leadership	3.01	0.35	2.89
Tolerance for ambiguity	2.68	0.19	2.61

*Significance level: p < .05*

At the same time, conflict management and leadership skills are rated much higher than average. In this test, leadership according to palliative care situations is a reflection of the ability to adequately lead and stimulate interaction and collaboration and to act as an active and positively receptive party to dialogue. This ability had the highest mean score (3.22) but also had the widest standard deviation (0.4), indicating that respondents’ responses on this scale were more varied than on the other scales.

Based on the results presented in Table 2, one cannot speak of any statistically significant correlations between the age and experience of the examinees and the demonstrated indicators of the scales “Intercultural Sensitivity”, “Intercultural Communication”, and “Intercultural Relation Building”. These three scales within this test reflect a greater extent the specificity of transcultural communication skills and readiness both to demonstrate appropriate behavior and to learn or develop it in this direction. Regardless of age or personal experience with palliative care, nurses show relatively average scores on these scales (2.31, 2.18, and 2.59, respectively).

The strongest and most unambiguous correlation is simultaneously observed between age and experience and the “Tolerance for Ambiguity” scale, which reflects readiness and receptivity in situations of ambiguity or uncertainty (0.816 and 0.782, respectively). A less strong correlation, which might qualify as a medium strength relationship, is determined between the “Conflict Management” and “Leadership” scales, with the strength of the relationship with the “Leadership” scale values being even stronger with age and work experience (0.477 and 0.412, respectively).

In general, these results can be considered expected, because, with age and experience in the medical field, particularly in palliative care, one develops experience in managing a variety of situations, develops confidence and ability to deal with different situations, and readiness to face situations that are little foreseeable. In the case of palliative care, situations of ambiguity or uncertainty in decision-making are fairly common, given the predominantly poly-morbid nature of the illness, the complex pattern of symptoms, and the emotional background associated with ongoing physical or mental suffering or the possibility of it [45].

Palliative care patients’ relatives’ rating on a five-point Likert scale of the importance for them of health care staff taking into account their culture while in palliative care showed a score of 4.74, with a mean deviation of 0.19. This indicates that for most patients’ relatives, cultural sensitivity in palliative care is very important and they consciously rate it highly.

## Discussion

To create quality care for palliative patients in a culturally sensitive way, it is important to understand what specific behavioral aspects a particular culture includes (social, ethnic, economic, etc.). The next step is to overcome prejudice, that is, to accept the patient as they are. Staff should have an in-depth knowledge of the cultural and historical characteristics of a group to which a patient belongs, and adequately assess the possible impact of these characteristics on the care process [46].

An analysis of medical education programs in the Russian Federation revealed insufficient or absent classes on the study of national peculiarities. Additionally, nurses had no practical experience in caring for patients from different cultural communities during their training. The material presented allowed implementing of the principle of an integrated and individual approach, highlighting the most important aspects in the training of staff for quality care of palliative patients. The theory of intercultural competence in palliative care was developed to help nursing staff care for patients of different cultures, different from their own.

Transcultural training for palliative care staff combines such concepts as cultural diversity, cultural traditions, cultural competence, and sensitivity [19, 47]. Furthermore, according to clinical psychologists, care and culture are inseparable. Pharmacotherapy without patient care is clinically ineffective, and care itself is a combination of knowledge of different values and behaviors specific to the ethnic culture of the patient.

A study of patients’ perspectives on the nurse-patient relationship conducted in Southern Ontario demonstrates two ways in which interactions in this tandem are carried out, the so-called “bright” and “dark” relationships. “Bright” relationships were judged to be those that involved an emotional response from the nursing staff, and conversely, “dark” relationships were judged to be those in which close emotional contact could not be created, with both patients and nursing staff constantly trying to avoid each other, resulting in feelings of mutual neglect. The outcome of this work was the experience that allowed us to evaluate, first of all, the value of nurse-patient relationships [48].

Other studies on the need to build trusting relationships with patients have demonstrated several ways of building trust. One group of patients claimed that the nursing staff, through attentiveness, competence, and the ability to create comfort, were able to build trust through certain character traits, which even contributed to the reduction of pain syndrome. Another group of patients argued about building trust by receiving honest information [34].

A study by Robbins and Davidhizar [49] highlighted the emotional aspect in the development of a close trusting relationship and nursing management. Several other researchers emphasized above all the importance of maintaining a sense of humor, especially in male patients, as they cannot always show openly their fear of illness. This study demonstrated that humor is a certain protective mechanism for male palliative patients [50].

Academic research offers increasing evidence that transcultural care plays an important role in the mechanism of creating quality care for palliative patients. The experience of such improvement is demonstrated by many countries and it can be transferred to Russia. At the same time, allowing patients to choose their preferred option of care is crucial. The coordination and organization of a multidisciplinary team in palliative care vary from country to country, depending on many factors (society, finances, traditions, religion, etc.). Some are developing a predominantly inpatient service (palliative units or inpatient beds, hospices), while others, on the contrary, are developing a home care service. Several studies have shown that between 50% and 70% of people with an incurable condition expressed a preference to end their lives in the home setting. For example, the detailed analysis conducted in the U.S. found that the rate of death in the home ranged from 18 to 32%. However, experience in many countries shows that more highly specialized care combines a variety of staff (inpatient medical staff and staff working directly with the public) [19, 51, 52].

The development of modern technologies and new drugs allows for successfully controlling pain. Communication skills and the creation of trusting relationships have a positive effect on several criteria (reduction of psychosomatic effects, normalization of biochemical indicators, etc.). Thus, improving the communication skills of nursing staff with patients can be achieved by recording (audio, video) conversations, followed by a discussion with professional psychologists and other specialists, especially in conditions of staff shortage among nursing staff [14, 50, 53–67].

The use of transcultural training of nurses, various methods of keeping palliative patients (at home, hospices, etc.), and the use of modern technologies improve the care of such patients and has a potentially significant positive impact on the outcomes of care. This, according to the definition of the WHO, constitutes palliative care, significant improvement in the quality of life of patients, satisfaction of their relatives, and improvement of relationships in society.

Modern medicine allows a high level of medical care, especially in the control of syndromes. Surveys conducted among relatives of deceased patients reveal quite a lot of insufficient knowledge in this area, which allowed detailing them and improving the quality of care provided to palliative patients. Based on the data obtained in this study, future research in this area may be aimed at determining the nurses’ preparedness for a more saturated multi-ethnic environment, including representatives of dozens of ethnic groups in need of palliative care. Increased workload through diversity may reduce the availability and effectiveness of care, which needs to be tested. The study also points to the importance of studying the readiness and preparation of relatives and caregivers of patients as an important factor in improving the quality of palliative care.

## Conclusions

It is known that the quality of life of any person consists of different aspects and depends on many factors. Nursing staff in the multinational society of the Russian Federation often encounter different ethnic groups and cultures in their work. Assessment of the importance of nursing staff training and the quality of transcultural care in palliative care provided at hospice and palliative care units (Moscow) demonstrates that there is still not enough time devoted to the educational aspect of providing medical care to palliative patients. The average scores on IRC scales related to intercultural sensitivity and communication appear to be lower than the average scores of the health care providers interviewed themselves. There is a correlation between participants’ age and experience and higher scores on the “Leadership” and “Tolerance for Ambiguity” scales. The main transcultural features in the work of nurses in adult palliative care departments are psychosocial support by cultural norms, taking into account cultural sensitivity, personal culture, and knowledge of patients’ language; of high importance is compliance with the gender norms of communication in the relevant culture. The modified transcultural approach with a justified model of high-quality medical services in palliative care for patients from other cultures shows that palliative care across cultural boundaries is paradoxical. The analysis of the findings highlighted the following aspects that a palliative nurse should possess: basic knowledge (a nurse’s general education and practical experience), knowledge of interpersonal relationship theory (theory of feeling one’s influence on others), diversity influences, and determinants (traditions and gender theory), and a patient’s health status (knowledge of problems that require urgent intervention). Other important aspects were the characterological qualities of a nurse - the ability to empathize and sympathize, to remain non-judgmental and accessible in trusting relationships, and highly communicative. The ability to listen properly will allow patients to feel their importance, get rid of fears and loneliness, and express needs and concerns. There has been no training on working with palliative patients of different ethnic groups in the preparation of medical nurses of different ages and work experience before. However, such training is in demand, its importance is highly valued by patients and is associated with improving the quality of medical care. The study holds practical significance in the sense that it calls for the development of novel nursing education programs that integrate the notion of transcultural values.

## Data Availability

Data will be available on request from the corresponding author.

## Abbreviations

IRC: Intercultural Readiness Check
